# Inguinal Hernia Containing Uterus, Fallopian Tube, and Ovary in a Premature Newborn

**DOI:** 10.1155/2015/807309

**Published:** 2015-08-16

**Authors:** Kıvılcım Karadeniz Cerit, Rabia Ergelen, Emel Colak, Tolga E. Dagli

**Affiliations:** ^1^Department of Pediatric Surgery, School of Medicine, Marmara University, 34899 Istanbul, Turkey; ^2^Department of Radiology, School of Medicine, Marmara University, 34899 Istanbul, Turkey

## Abstract

A female infant weighing 2,200 g was delivered at 34 weeks of gestation by vaginal delivery. She presented with an irreducible mass in the left inguinal region at 32 days of age. An ultrasonography (US) was performed and an incarcerated hernia containing uterus, fallopian tube, and ovary was diagnosed preoperatively. Surgery was performed through an inguinal approach; the uterus, fallopian tube, and ovary were found in the hernia sac. High ligation and an additional repair of the internal inguinal ring were performed. Patent processus vaginalis was found during contralateral exploration and also closed. The postoperative course was uneventful. After one year of follow-up, there have been no signs of recurrence.

## 1. Introduction

Indirect inguinal hernia is the most common congenital anomaly of infancy and childhood with an incidence ranging from 0.8% to 4% [1]. It is seen more often in the first year of life. In premature infants, the incidence increases to 30%. In female infants, sliding inguinal hernias mostly contain the ovary with or without fallopian tube. The presence of the uterus within the hernia sac (hernia uterus inguinale) and incarceration of the adnexa of the uterus are an extremely rare condition in infants [2]. Since only a few cases are described in literature, we herein report a premature female infant who had an inguinal hernia containing uterus, fallopian tube, and ovary.

## 2. Case Report

**Figure 1:**
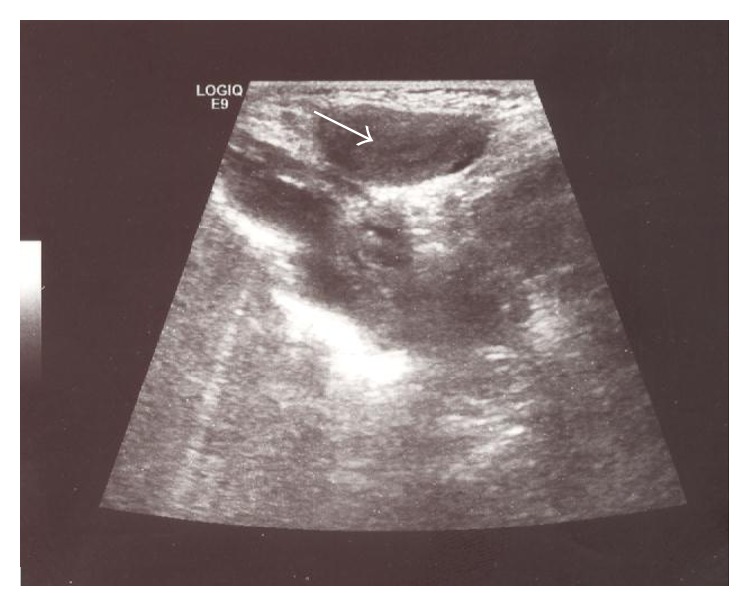
Ultrasonography image: a solid mass in right inguinal channel with a clearly visible endometrial lining.

**Figure 2:**
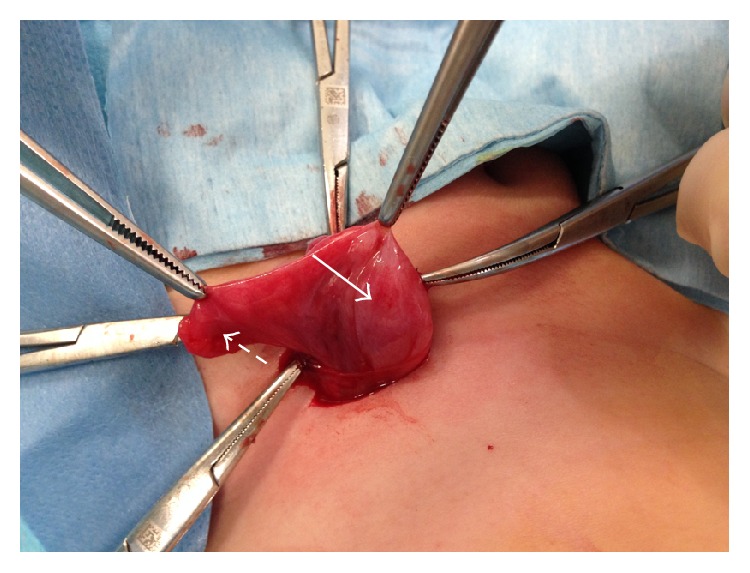
Intraoperative findings. Hernia sac containing uterus, fallopian tube, and ovary.

A female premature infant was delivered at 34 weeks of gestation (birth weight 2,200 g, height 44 cm, and Apgar score 7/9) by vaginal delivery. No inguinal masses were noted and her external genitalia appeared normal during her first examination. She was referred to the pediatric surgery unit at 32 days of age (weight 3150 g, height 48 cm), with an irreducible mass in the left inguinal region, noticed by her pediatrician a few hours ago. There was no history of irritability, pain, erythema, or vomiting. On physical examination, the patient had an irreducible, soft mass in the left inguinal region. An ultrasonography (US) was performed because an incarcerated ovarian hernia was suspected. A solid mass in left inguinal channel with a clearly visible endometrial lining was seen and an incarcerated hernia containing uterus, fallopian tube, and ovary was diagnosed preoperatively (Figure 1). Surgery was performed through an inguinal approach; the uterus, fallopian tube, and ovary were found in the hernia sac (Figure 2). The organs were freed from the hernia sac. Gentle and careful dissection was required due to strong adhesions between the organs and the hernia sac, which was very thin. The organs were edematous but perfusion appeared normal. The reduction of the hernia contents into the abdomen through the inguinal canal was slightly difficult. A high ligation and an additional repair of the internal inguinal ring were performed to prevent recurrence. During contralateral exploration, a patent processus vaginalis was found and repaired. The postoperative course was uneventful. At one-year follow-up, the patient had neither clinical nor radiological evidence of a recurrence. Pelvic organs appeared normal and in the correct location.

## 3. Discussion

The current case is a 32-day-old female premature infant that presented with an irreducible indirect inguinal mass. The uterus, fallopian tube, and ovary were identified within an inguinal hernia sac. The hernia contents were reduced into the abdomen through the inguinal canal and a high ligation plus additional repair of the internal inguinal ring were performed.

Processus vaginalis develops at around the sixth month of fetal growth as an evagination of parietal peritoneum. Depending on gender, it is accompanied by the testis or round ligament of the uterus and passes through the inguinal canal up to the scrotum or labium major. Processus vaginalis is relatively small in female infants and obliterates around eight months of gestation. If patency persists, it is termed the canal of Nuck [2].

Inguinal hernia containing an ovary with or without a fallopian tube is not uncommon in female infants. However, an inguinal hernia containing the uterus is extremely rare. The etiology of this pathology is controversial. An anatomic abnormality with primary weakness of the uterine and ovarian suspensory ligaments is suspected. Thomson offered the hypothesis that if there is failure of fusion of the Mullerian ducts leading to excessive mobility of the ovaries plus nonfusion of the uterine cornua, the chance of herniation of the entire uterus, ovary, and fallopian tube into the inguinal canal is increased [3]. On the other hand, Fowler theorized that elongated ovarian suspensory ligaments were the primary cause or the secondary effect of a hernia [4]. The finding of an anatomic abnormality may compromise fertility; therefore, careful gynecologic follow-up is required until the childbearing age.

The presence of the uterus in an inguinal hernia in boys is attributed to the persistence of Mullerian duct derivatives. Male pseudohermaphroditism is characterized by the presence of Mullerian duct derivatives (uterus, cervix, fallopian tubes, and upper third of the vagina) in phenotypic male patients [5]. Because of the normal female phenotype, analysis of the chromosomes was not performed in the present case.

Due to the rarity of these cases where an indirect hernia sac contains the uterus, fallopian tube, and ovary, different aspects of surgical treatment must be kept in mind. Some authors perform a classic herniorrhaphy with a high ligation through an inguinal approach, while some authors advocate additional closure of the internal ring, as performed in our patient [2]. Suzuki et al. reported one pediatric case with recurrence after reduction of the hernia contents into the abdomen and ligation of the internal ring under laparotomy, in which a high ligation and repair of the inguinal canal under an inguinal approach were performed [6]. Okada et al. recommended simple herniorrhaphy for indirect inguinal hernia containing the uterus, bilateral ovaries, and fallopian tubes [7]. The surgical procedure for inguinal hernia containing uterus is quite different from the cases containing only the ovary as these organs are strongly attached to the hernia sac and it is difficult to free them from the wall of the hernia sac. After freeing these attachments without damaging the organs, we recommend high ligation and additional repair of the internal inguinal ring to prevent recurrence. Furthermore, we also recommend contralateral exploration to prevent the infant from another operation.

Because of the risk of damaging herniated structures during the surgical procedure, a careful preoperative investigation is necessary. US should be routinely performed in female infants with an irreducible palpable inguinal mass [7, 8]. US is an accurate and easily available choice for diagnosis. Preoperative US using a high-frequency transducer is therefore very helpful in reaching a diagnosis with an efficacy considered to be almost 100% [9]. Early recognition by a pediatric surgeon or a neonatologist assures prompt surgical intervention and prevents the injury to the herniated organs in incarcerated inguinal hernias containing uterus, fallopian tube, and ovary.

## 4. Conclusion

When an atypical inguinal hernia is diagnosed in a premature female infant, we advise prompt ultrasonography in all cases. Early surgical intervention is necessary to prevent the damage of herniated organs, because unexpected reproductive structures may be involved in the hernia sac.
